# Traumatic Pseudoaneurysm of the Middle Meningeal Artery Causing an Intracerebral Hemorrhage

**DOI:** 10.1155/2010/219572

**Published:** 2010-06-10

**Authors:** Wellingson Silva Paiva, Almir Ferreira de Andrade, Robson Luis Amorim, Eberval Gadelha Figueiredo, Manoel Jacobsen Teixeira

**Affiliations:** ^1^Division of Neurosurgery, Department of Neurology, Hospital Das Clinicas, University of Sao Paulo Medical School, 05406000 Sao Paulo, Brazil; ^2^Neurosurgical Emergency Unit, Division of Neurosurgery, Department of Neurology, Hospital Das Clinicas, University of Sao Paulo Medical School, Eneas Aguiar Street, Number 255, 4th floor, 05403010 Sao Paulo, Brazil

## Abstract

Traumatic aneurysms comprise less than 1% of all intracranial aneurysms. Most of these aneurysms are actually false aneurysms, or pseudoaneurysms, which are caused by the rupture of entire vessel wall layers, with the wall of the aneurysm being formed by the surrounding cerebral structures. Traumatic pseudoaneurysms of the middle meningeal artery are also rare. Only four cases have been reported in the literature with intracerebral hematoma. In this paper, the authors report a case of a patient with a ruptured traumatic pseudoaneurysm of the MMA who presented with an intracerebral hematoma in the left temporal region immediately after trauma; the patient underwent endovascular treatment.

## 1. Introduction

Traumatic aneurysms of the middle meningeal artery (MMA) are uncommon and are a well-known cause of intracranial hemorrhage (ICH). Traumatic aneurysms comprise less than 1% of all intracranial aneurysms [1]. Most of these aneurysms are actually false aneurysms, or pseudoaneurysms, which are caused by the rupture of entire vessel wall layers, with the wall of the aneurysm being formed by the surrounding cerebral structures [2]. Traumatic pseudoaneurysms of the middle meningeal artery (MMA) are also rare. Acute or delayed epidural hematoma is the most frequent presentation of traumatic pseudoaneurysms [2, 3], nonetheless they may sometimes be associated with a subdural hematoma or subarachnoid hemorrhage [2, 3]. 

Intracerebral hematoma due to ruptured traumatic pseudoaneurysm of the MMA is extremely rare [4]; only four cases have been reported in the literature, and our case is the first application of specific diagnosis technique. The authors report a case of a patient with a ruptured traumatic pseudoaneurysm of the MMA who presented with an intracerebral hematoma (ICH) in the left temporal region immediately after trauma; the patient underwent endovascular treatment.

## 2. Case Report

**Figure 1:**
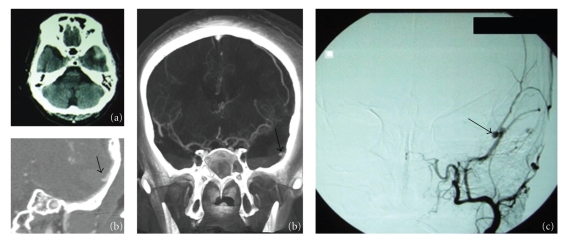
Patient with traumatic intracerebral hematoma: in (a) skull computed tomography with temporal hematoma, in (b) angiotomography with hematoma and pseudoaneurysm, and in (c) digital angiography confirms a pseudoaneurysm in middle meningeal artery.

A 33-years-old man suffered blunt head trauma in an automobile accident and was admitted to a local neurosurgical hospital. On admission, the patient was drowsy and his Glasgow Coma Scale (GCS) score was 13/15. Both pupils were isocoric and reactive to light. Neurological exam revealed no further abnormalities. Head Computed Tomography (CT) scan revealed a small intracerebral hematoma in the left middle cranial fossa associated with temporal fracture (Figure 1(a)). A multislice angioCT scan revealed a pseudoaneurysm of meningeal middle artery underneath temporal fracture (Figure 1(b)). Sequentially, the patient was submitted to cerebral angiogram that revealed active bleeding from the left MMA and an active extravasation of contrast media, and a pseudoaneurysm with 0.3 cm in diameter, that had arisen from the anterior branch of the left MMA (Figure 1(c)). Embolization of MMA with Hystoacryl was performed to complete obliteration of the artery. Follow-up head CT scan showed no significant hematoma enlargement. The patient presented an improvement in consciousness for GCS score 15 after two days. On discharge, four days after trauma his GCS score was 15.

## 3. Discussion

Hematomas by ruptured traumatic pseudoaneurysms of MMA usually present with extradural hematomas [5, 6]. However, traumatic pseudoaneurysms of the MMA, although rare, have been considered as a possible etiology of acute intracerebral hematoma. Only four cases of ICHs caused by the rupture of traumatic MMA pseudoaneurysms have been reported in the English literature [7–10]. In this paper, we describe the first application of angioCT for diagnosis of pseudoaneurysm of MMA.

In 92% of cases, traumatic aneurysms are associated with a skull fracture extending across the course of the meningeal artery and causing a tear in the arterial wall. Histologically, they are false aneurysms that contain none of the normal arterial layers but are lined by fibro-connective tissue surrounding a hole in the arterial wall [11].

On angiography, meningeal pseudoaneurysms have specific characteristics [12, 13]; they are peripherally located at a distance from a branching point and do not show evidence of a neck. The sac is sometimes irregular. Its filling and emptying are delayed and very slow, so that the contrast medium has time to settle at the bottom of the aneurysm and the pre- and postaneurysmal segments of the meningeal artery are not opacified at the same time. In our case, the diagnosis was realized by CT, and the angiogram performed for confirm the diagnosis and to treat the aneurysm. The findings in angiogram indicate that brain hematoma arises to pseudoaneurysm of MMA. 

The natural history of traumatic aneurysms is not well known, but progressive growth of traumatic aneurysms has been demonstrated on repeated angiograms [14, 15]. Traumatic PMMA may regress, thrombose, enlarge, or rupture. Pseudoaneurysm of the middle meningeal artery carries a high risk of rupture, producing an abrupt neurological deterioration due to intracranial hematoma after a 3- to 30-day interval [16], which is associated with a mortality of 50% [16, 17]. Therefore, preventive therapy is required for this vascular lesions [17, 18]. Because rupture of a pseudoaneurysm of the middle meningeal artery can be lethal, like this case, we emphasize early diagnosis and early preventive treatment.
